# The press-fit technique without screws and bone graft can be used as an alternative method in Crowe type II and III hip dysplasia

**DOI:** 10.55730/1300-0144.5712

**Published:** 2023-08-11

**Authors:** Aziz ÇATALTEPE, Yusuf İYETİN

**Affiliations:** 1 Department of Orthopedic Surgery and Traumatology, Medipol University, İstanbul, Turkiye; 2 Department of Orthopedic Surgery and Traumatology, Pendik Hospital, İstanbul, Turkiye

**Keywords:** Total hip arthroplasty, developmental dysplasia of the hip, press-fit technique, medialization, small cup

## Abstract

**Background/aim:**

Developmental dysplasia of the hip (DDH) is the most common cause of secondary hip arthrosis. The primary purpose of this study was to assess the results of an oversized hemispherical cup via the press-fit technique used for Crowe type II and III DDH without screws and to determine if adequate medialization and initial stability of the acetabular component would allow us to avoid screw and graft use.

**Materials and methods:**

Between February 2012 and May 2020, the current study analyzed 43 hips with Crowe type II and III DDH treated with a porous-coated cup by placing the press-fit technique or screw. The acetabular cup was fixed with the press-fit technique without additional screws in 27 hips and with screws in 16 hips. The inclusion criterion in this study was a minimum 2-year-period after the surgery.

**Results:**

The mean duration of follow-up was 6.83 **±** 2.67 years in the press-fit group and 6.21 **±** 2.01 years in the screw group. The mean age of the patients was 47.96 **±** 12.37 years in the press-fit group and 50.5 **±** 12.37 years in the screw group. Measurements revealed that the hip center of rotation (HCR) was located more medially and superiorly postoperatively than preoperatively in both groups. The mean cup coverage in the screw group was 85.58% **±** 7.51% (75.3%–97.2%), while it was less than 90.41% **±** 6.15% (76.3%–98.2%) in the press-fit group (p = 0.038). No component was revised because of loosening, and all of the implants were radiologically stable within the observation period. No statistically significant differences were observed regarding the postoperative limp-length discrepancy between the groups (p = 0.496).

**Conclusion:**

Press-fit implantation of a porous-coated acetabular component without screws can also be used as an alternative method for THA in Crowe type II and III DDH. The initial stability was obtained using the press-fit technique with a small cup positioned more medially and superiorly, which may allow the surgeon to avoid screw and graft use.

## 1. Introduction

Developmental dysplasia of the hip (DDH) is the most common cause of secondary hip osteoarthritis, especially in younger patients [1–3]. Although total hip arthroplasty (THA) is an efficacious treatment for advanced hip arthritis, THA for the reconstruction of the acetabulum presents specific problems such as insufficient acetabular cup bone coverage, an abnormal hip center, and abnormal anatomy of neurovascular structures in patients with DDH [3–6].

It is evident that a superolateral bone defect is frequently observed above the surface of the acetabular component at the level of the true acetabulum and contracted soft tissue that may not allow placing the acetabular component in a reasonable region of the hip, particularly in hips with Crowe II and III DDH [7–9]. Several studies have indicated that inaccurate acetabular component placement may trigger postoperative complications, including dislocation, increased wear rates, a high revision rate, and leg-length discrepancy [8–12]. In contrast, some other studies have reported that the placement of the acetabular component far from the true acetabulum might provide better bone coverage than the anatomic position, especially in hips with Crowe types II and III [13–16]. Nawabi et al. performed a study with 27 Crowe type II and III hips treated with a high hip center (HHC) [16]. Their study demonstrated that there were no revisions for acetabular component loosening, and there was no significant difference between the HHC group and the anatomic hip center group in terms of Kaplan-Meier survivorship for all-cause revisions.

The initial stability of a cementless acetabular component is crucial because insufficient primary cup fixation may lead to early loosening [17, 18]. Various reconstruction methods to obtain initial stability of the acetabular cup in patients with DDH have been described, including the press-fit technique without screws, using screws, a structural bone graft, and a small cup placed in an anatomical location or a superior position [19–21]. Moreover, a component can be placed using the medialization technique to achieve satisfactory bony coverage in DDH [22, 23]. In the last 2 decades, several studies have reported favorable outcomes of THA with the press-fit technique in patients with nondysplastic hips; however, few reports have described the results of uncemented acetabular components implanted into DDH by applying press-fit fixation without screws [24–26]. Specifically, little is known about the durability of the acetabular cup in conjunction with the press-fit-only technique without bone graft in Crowe types II and III.

Crowe type I is mainly desscribed as primary degenerative osteoarthrosis of the hip joint [10]. Crowe type IV hips have severely deficient acetabular bone stock, and femoral shortening osteotomy is often needed to place the acetabular cup near the true acetabulum region owing to severe luxation of the femoral head [10]. Therefore, the current study evaluated patients who underwent THA and were diagnosed with Crowe II and III DDH in our clinic. The primary purpose of the study was to assess the results of an oversized hemispherical cup via the press-fit technique used for Crowe type II and III DDH without screws and determine if adequate medialization and initial stability of acetabular components would allow us to avoid screw and graft use.

## 2. Materials and methods

### 2.1. Study design and data collection

The current study retrospectively analyzed 43 consecutive hips treated with THA by applying the press-fit technique or screws between February 2012 and May 2020. All of the hips had Crowe type II or III DDH. The hips were analyzed for clinical and radiographic results using the database of our institution.

Inclusion criteria were a minimum 2-year-period after surgery and Crowe type II or III DDH in each group. Four hips that were initially placed superiorly more than 15 mm from the anatomic hip center of rotation (HCR) were excluded from the study. Two hips were lost to follow-up. One patient with Crowe type II DDH died of causes unrelated to the surgery. A total of 43 primary total hip replacements that met the inclusion criteria were assessed. The hips were divided into 2 groups according to the fixation method.

All of the operations were performed with the same operative technique using a posterolateral approach and the same surgeon. All of the procedures were performed using the same type of cementless acetabular cup (Trilogy, Zimmer, Warsaw, Indiana). The component has supplemental screw fixation holes, none used in the press-fit group. A highly crosslinked polyethylene liner or ceramic liner was applied in all of the operations. One type of prosthetic head was a ceramic head used on every hip. The acetabulum was reamed with an initial reamer of 36 mm to maximize the depth of the acetabular cavity and the bony coverage of the acetabular cup. Acetabular reaming was deepened until the medial wall was exposed. A trial shell that was 1 or 2 sizes larger than the final reamer was then inserted. The cup was positioned as medially as possible. The acetabular component was inserted with press-fit fixation in the press-fit group. Additional screws were applied when the acetabular cup was needed to reinforce the initial stability of the cup in the screw group. Additional structural bone grafts, greater trochanter osteotomies, and subtrochanteric femoral shortening osteotomies were not performed in any of the hips during surgery. Immediate full weight bearing was allowed using crutches during the early postoperative rehabilitation period.

### 2.2. Radiological assessment

Radiographic measurements were performed preoperatively, immediately after the operation, at 4 weeks, at 3, 6, and 12 months, and then at a yearly visit. A picture archiving and communication system (PACS) was used to evaluate the radiographic measurements. In unilateral DDH, the anatomic hip center was measured by contrast with the contralateral hip. In bilateral DDH, the anatomic hip center was calculated using a method described by Ranawat et al. [27]. The approximate center of the femoral head (ACFH) was used as the reference point to calculate the distance to the center of the prosthetic femoral head (CPFH). The dislocation’s severity was assessed using the classification of Crowe et al. [28]. Radiolucent gaps on the initial postoperative radiograph and radiolucent lines or osteolysis at the bone component interface on the final follow-up radiographs were identified in the 3 zones described by De Lee and Charnley [29]. Cup migration and changes in the abduction angle were measured using the technique of Calaghan et al. [30]. A horizontal or vertical difference in position of 3 mm or more and a change in abduction angle of at least 5° or a rotational change of ≥8° were considered migration. The formula described by Spangehl et al. was used to calculate the percentage of an acetabular component covered by intact native acetabular bone on the anteroposterior (AP) pelvis radiograph [20].

The Harris Hip Score (HHS) was used to evaluate the functional outcomes before surgery and at the last follow-up.

### 2.3. Statistical analysis

Number Cruncher Statistical System (NCSS) 2007 Statistical Software (Utah, USA) was used for the statistical analysis. Nominal data were expressed as frequencies or percentages, and quantitative data were expressed as the mean ± standard deviation. The Shapiro-Wilk test was performed to test for normality. The paired t test was used to analyze the pre- and postoperative HCR-H, HCR-V, limp-length discrepancy, and differences in the pre- and postoperative HHS. Groups were compared using the independent t test for normally distributed continuous variables. The chi squared test was used to analyze qualitative comparative parameters. P < 0.05 was considered statistically significant.

## 3. Results

The acetabular cup was fixed with the press-fit technique without additional screws in 27 hips (mean follow-up 6.83 **±** 2.67 years) and with screws in 16 hips (mean follow-up 6.21 **±** 2.01 years). In the classification of the hips, 23 were type II, and 20 were type III. The mean age of the patients was 47.96 **±** 12.37 years in the press-fit group, and it was 50.5 **±** 12.37 years in the screw group. The mean duration of the operation in the screw group (91.93 **±** 8.81 min) was significantly longer than that in the press-fit group (81 **±** 10.35 min) (p = 0.002). No significant differences were observed in terms of the mean size of the acetabular component between the groups (p = 0.031) (Table 1).

**Figure 1:**
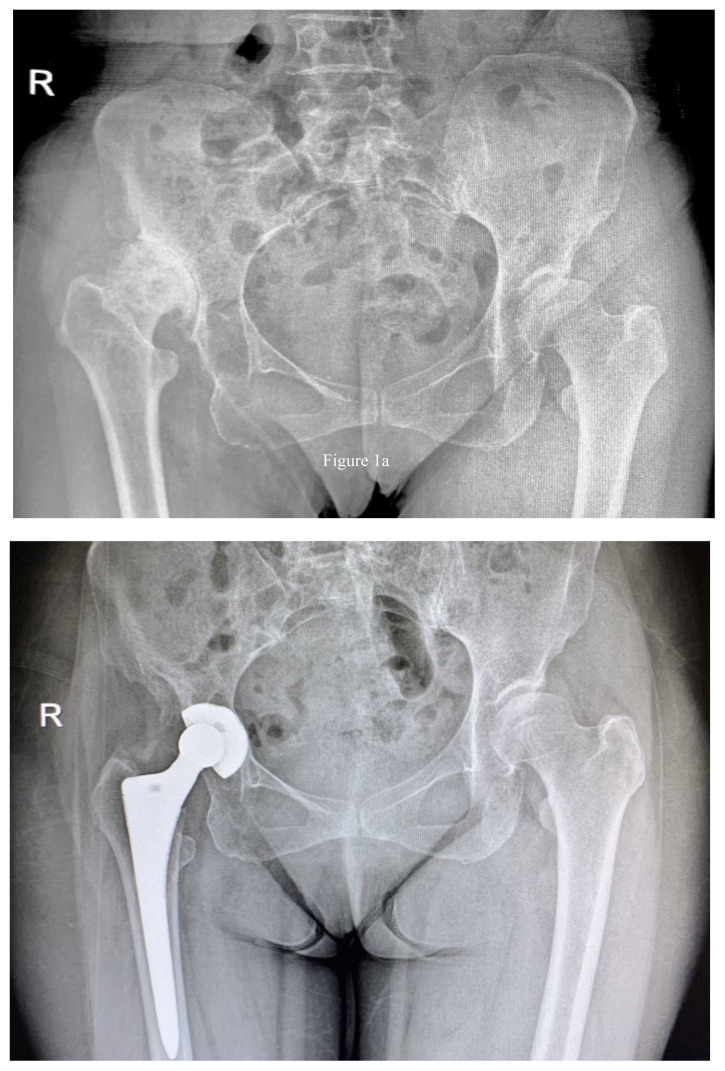
(a) Preoperative X-ray of a Crowe type II hip, (b) postoperative X-ray of THA taken 3.1 years later. The right hip was treated with the press-fit technique.

**Figure 2:**
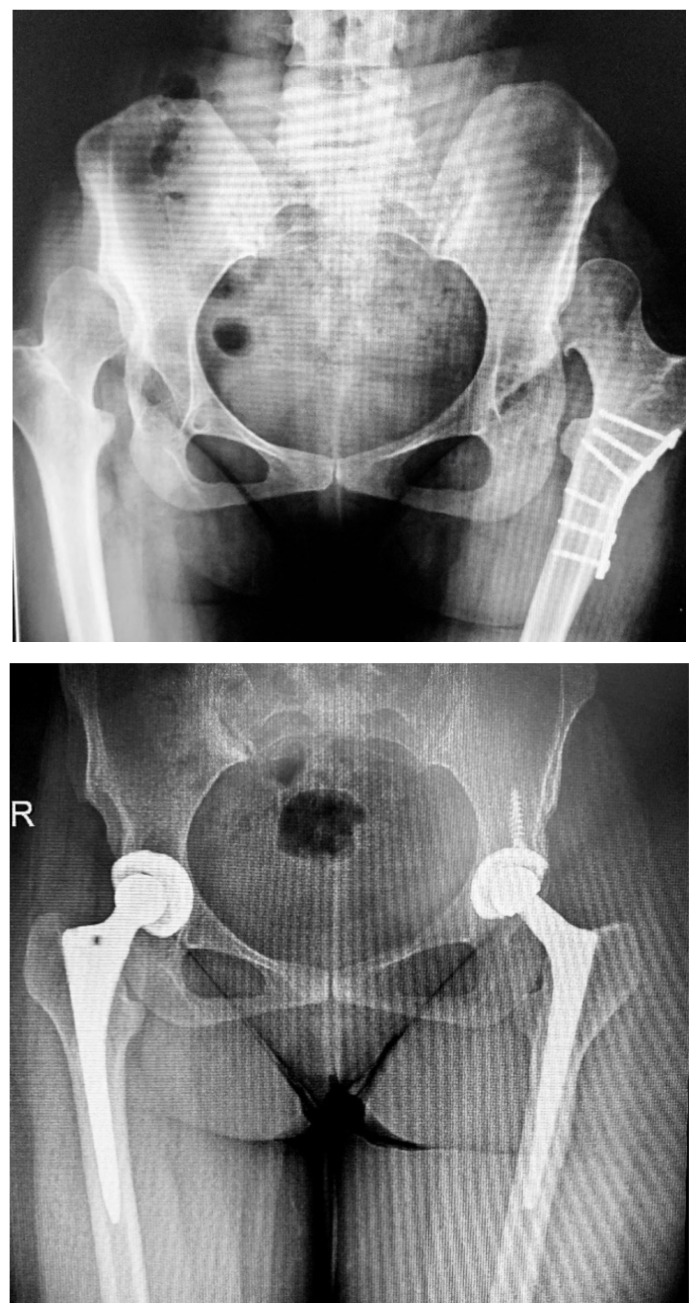
(a) Preoperative X-ray of bilateral Crowe type III hips. (b) Postoperative X-ray of bilateral THA taken 6.8 years later. The right hip was treated with the press-fit technique, while the left hip was fixed with screws.

No statistically significant differences were found regarding the mean postoperative anteversion and inclination of the acetabular shell (p = 0.724 and p = 0.796, respectively) (Table 2). There were statistically significant differences between the preoperative and postoperative HCR-horizontal distance (HCR-H) and HCR-vertical distance (HCR-V) in both groups (Table 3; Figures 1a, 1b, 2a, and 2b). The measurements revealed that the HRC was located more medially and superiorly postoperatively than preoperatively in the combined the 2 groups (Table 4). A significant difference was found between Crowe types II and III regarding the cup sizes, cup coverage, and the mean ACFH-CPFH distance (Table 5).

The mean cup coverage in the screw group was 85.58% **±** 7.51% (75.3%–97.2%), which was less than in the press-fit group, at 90.41% **±** 6.15% (76.3%–98.2%) (p = 0.038). Nonprogressive radiolucent lines occurred in zone I in 1 hip and in zone III in 1 hip in the press-fit group. Also in the press-fit group, 1 hip had radiolucent lines involving zones I and II. Nonprogressive radiolucent lines in the screw group were observed in zone I in 2 hips and zone III in 1 hip. No revisions, loosening, or dislocation occurred during the follow-up in either group.

No statistically significant differences were observed regarding the postoperative limp-length discrepancy between the groups (p = 0.496) (Table 2).

The postoperative HHS improved significantly at the last follow-up compared with the preoperative HHS in both groups. However, no significant differences were observed regarding the postoperative HHS between the groups (p = 0.390) (Table 1).

One patient experienced a pulmonary embolism treated with low molecular weight heparin (LMWH). In 1 hip (1.31%), a 54-year-old female experienced minimally displaced intraoperative fractures of the proximal part of the femur during insertion of the stem, which required osteosynthesis with 2 cable wires, in the screw group. This hip was evaluated for 2.9 years, and there was no loosening for any reason. One hip encountered a superficial surgical wound infection treated with antibiotics in the screw group. None of the hips had a deep infection.

## 4. Discussion

Acetabular cup coverage is vital in making preoperative and postoperative decisions regarding cup placement and additional support [4, 6]. Various techniques, such as bone graft reconstruction and small cups, can be used to tackle inadequate bone coverage of the acetabular cup [8, 23, 31–33]. A femoral head autograft might be needed in some hips where the uncoverage is more than 30% [20, 21, 34]. Nevertheless, several studies suggested not using bulk autografts owing to cup loosening, mechanical failure, increased operative time, the need for more soft tissue exposure, and concerns for graft resorption [33, 35, 36]. It has been suggested that at least 70% osseous cup coverage by native bone for sufficient stability during surgery should be obtained [36, 37]. Anderson et al. confirmed that bulk autogenous grafting is not needed to adequately fixate acetabular cups when at least 70% of the cup is covered by host bone [31]. In the present study, all of the cups were covered by host bone with a mean of 90.41 **±** 6.15 in the press-fit group and 85.58 **±** 7.51 in the screw group; therefore, no femoral head autograft or allograft was applied in any of the hips.

The acetabular component position plays a crucial role in the long-term results of THA for patients with DDH [38]. Recent clinical studies have reported that excellent results, adequate cup coverage, and correct inclination in DDH treated with uncemented THA can be obtained by placing the acetabular component more medially and slightly superiorly [6, 12, 16, 22, 23, 33]. Dorr et al. performed a study with 24 hips in which the acetabular cup was placed beyond the Kohler line and followed-up for 7 years [23]. They concluded that the medial protrusion technique might allow better fixation of the acetabular cup and reduce the use of bone grafts and excessive polyethylene wear. Watts et al. retrospectively analyzed 88 hips with Crowe II and III dysplasia and found that 70 of 88 hips placed at the level of the anatomic hip center did not show any signs of loosening or revision [15]. However, 18 of the 88 hips placed far from the native hip center showed loosening measurements and were revised due to the loosening of the cups. Xu et al. measured the 3-dimensional coverage postoperatively in 45 hips, with Crowe type II in 17 hips and Crowe type III in 28 hips [4]. In their study, 30 hips were treated with the press-fit technique, while 15 hips were fixed with screw fixation. They positioned the acetabular cup at the anatomical HCR and did not use a structural bone graft, but a particulate bone autograft was used in 33 hips. The cup coverage by native host bone for the press-fit group was 73.88 **±** 10.75%, and for the screw group, it was 71.19 **±** 11.43%. Conversely, an acetabular component placed in a slight verticalization of the HCR without lateral displacement can provide better bone coverage than an anatomic position, especially in patients with Crowe type II and III DDH [1, 6, 14, 16, 33]. Sakemi et al. used computer software and evaluated bone coverage in 32 patients with unilateral Crowe type II and III DDH [33]. The study showed that superior cup placement obtained more cup coverage by the host bone, which was 87.5% at an HCR-V of 25 mm. The present study obtained the same outcomes. Herein, it was attempted to position the acetabular component in or near the anatomic acetabular region. The HRC was located more medially and superiorly than the preoperative HCR in the combined group. In the current study, the slight elevation of the hip center with medialization and without lateralization showed satisfactory clinical and functional outcomes in both groups. The outcomes of our research revealed that there was no loosening or revision during the follow-up period.

The initial stability of the cementless acetabular component is crucial because insufficient primary cup fixation may lead to early loosening [17,18]. In a recent study, Du et al. suggested that more than 75.5% cup coverage values with or without screws can provide sufficient stability in patients with Crowe II and III DDH [8]. The same conclusions were drawn by Tikhilov et al. in 2016. Based on the current study, we agree that press-fit fixation can be applied by reaching at least 76.3% cup coverage by host bone [39]. Moreover, Hartofilakidis et al. suggested that a 40- to 42-mm cup may permit the surgeon to use press-fit fixation by providing at least 80% coverage of the acetabular component with bone [32]. Takao et al. performed a retrospective study with 96 patients treated with the press-fit-only technique [26]. They obtained successful outcomes with no revisions during a minimum 6-year follow-up. However, they did not exclude Crowe type I, which their cases mainly consisted of. In addition, a morselized bone graft was used in selected cases. The mean cup diameter was 50 mm in their study. However, Crowe type I was excluded, and bone grafts were not applied in the present study. The current study achieved more cup coverage owing to smaller cups and more medialization than their results.

The present study had some limitations. The main limitation was that it was a retrospective evaluation with a relatively small sample size. However, the patients in this study were identified from a consecutive series with DDH, which can diminish the possibility of selection. The other limitation was that the follow-up period of at least 6.8 years was relatively short for assessing the duration of THA. It was intended to follow all of the patients in the following years to address further surveys at a mean of 10 and 15 years postoperatively. Adequate stability of the acetabular components can provide favorable results in the long term. AP pelvic radiographs are primarily used to assess preoperative planning for the location of acetabular component placement and patient follow-up for DDH [2]. The present study performed only AP pelvic radiographs to evaluate the acetabular cups, which provide 2-dimensional information. Finally, the present study did not search for the presence of a postoperative limp, which was associated with multiple factors. Linear wear could have been focused on, because the mean inclination was more than was aimed for in both groups.

In conclusion, press-fit implantation of a porous-coated acetabular component without screws can also be used as an alternative method for THA in Crowe type II and III DDH. The initial stability was obtained using the press-fit technique with a small cup positioned more medially and superiorly, which may allow the surgeon to avoid screw and graft use.

## Figures and Tables

**Table 1:** Patient demographic data.

Variable	Press-fit group	Screw group	*p-value
Hips (n)	27	16	
Mean age (year)	47.96 ± 12.37	50.5 ± 12.37	0.545*
Sex (female/male)	25/2	15/1	0.439+
BMI (kg/m^2^)	24.87 ± 3.63	25.55 ± 3.91	0.592*
Side (right/left)	12/15	7/9	0.859+
Operation time (minute)	81.01 ± 10.35	91.93 ± 8.81	0.002*
Follow-up time (year)	6.83 ± 2.67	6.21 ± 2.01	0.463*
Crowe DDH classification			
Type II	18 (66.67%)	5 (31.25%)	0.023+
Type III	9 (33.33%)	11 (68.75%)	0.023+
Cup size (mm)	44.58 ± 3.71	42.14 ± 2.14	0.031*
Linear			
Ceramic	12 (44.44%)	3 (18.75%)	0.048+
Polyethylene	15 (55.56%)	13 (81.25%)	0.048+
Femoral head size (mm)	27.75 ± 4.31	24.57 ± 3.08	0.021*
HHS			
Preoperatively	40.58 ± 4.64	41.14 ± 5.07	0.731*
Postoperatively	88.58 ± 5.07	87.00 ± 5.97	0.390*
†p-value	0.0001	0.0001	

Values are given as the mean (standard deviation) or n (%), as appropriate, and the p-value was calculated using the independent t test (*), the chi squared test (+), and the paired test (†). BMI: body mass index, DDH: developmental dysplasia of the hip, HHS: Harris hip score.

**Table 2:** Postoperative radiograph data.

Variable	Press-fit group n = 27	Screw group n = 16	*p-value
Cup inclination angle	48.72 ± 5.14°	49.36 ± 5.77°	0.724*
Cup anteversion angle	13.88 ± 3.91°	14.25 ± 4.65°	0.796*
Cup coverage (%)	90.41 ± 6.15	85.58 ± 7.51	0.038*
Radiolucent line			
Zone I	2 (8.33%)	2 (14.3%)	
Zone II	1 (4.17%)	0	
Zone III	1 (4.17%)	1 (7.14%)	
Limp-length discrepancy (cm)			
Preoperatively	2.75 ± 0.74	3.13 ± 0.75	0.143*
Postoperatively	0.48 ± 0.25	0.54 ± 0.27	0.496*
†p-value	0.0001	0.0001	

Values are given as the mean (standard deviation) or n (%), as appropriate, and the p-value was calculated using the independent t test (*) and paired test (†).

**Table 3:** Changes in the HCR between the groups.

Variable		Press-fit group (n: 27)	Screw group (n: 16)	*p-value
HCR-H (mm)	Preoperatively	30.8 **±** 2.47	30.37 **±** 2.29	0.596*
	Postoperatively	26.05±2.08	25.61 **±** 1.88	0.520*
	†p-value	0.0001	0.0001	
HCR-V (mm)	Preoperatively	14.41 **±** 2.95	14.86 **±** 2.84	0.644*
	Postoperatively	20.89 **±** 4.58	22.97 **±** 5.03	0.200*
	†p-value	0.0001	0.0001	
Mean ACFH-CPFH distance (mm)		8.54 ± 2.38	10.22 ± 2.36	0.042*

Values are given as the mean (standard deviation) and the p-value was calculated using the independent t test (*) and paired test (†). HCR-H: hip center of rotation-horizontal distance, HCR-V: HCR-vertical distance, ACFH: approximate center of the femoral head, CPFH: center of the prosthetic femoral head.

**Table 4:** Overall results of combining the 2 groups.

Variable		Press-fit group + screw group n: 43
Shell (mm)		43.60 ± 3.40
Coverage (%)		88.63 ± 6.99
HCR-H (mm)	Preoperatively	30.65 ± 2.38
	Postoperatively	25.88 ± 1.99
	†p-value	0.0001
HCR-V (mm)	Preoperatively	14.58 ± 2.88
	Postoperatively	21.66 ± 4.80
	†p-value	0.0001
the mean ACFH-CPFH distance (mm)		9.16 ± 2.48

Values are given as the mean (standard deviation) and the p-value was calculated using the paired test (†).

**Table 5:** Changes in the HCR between Crowe type II and III.

Variable		Crowe type II n: 23	Crowe type III n: 20	*p-value
Cup size (mm)		45.3 ± 3.51	41.89 ± 2.22	0.001*
Coverage (%)		93.18 ± 4.47	83.57 ± 5.72	0.0001*
HCR-H (mm)	Preoperatively	30.67 ± 2.69	30.62 ± 2.07	0.957*
	Postoperatively	26.21 ± 2.15	25.52 ± 1.79	0.294*
	†p-value	0.0001	0.0001	
HCR-V (mm)	Preoperatively	13.3 ± 2.25	15.99 ± 2.89	0.003*
	Postoperatively	18.9 ± 3.45	24.72 ± 4.22	0.0001*
	†p-value	0.0001	0.0001	
the mean ACFH-CPFH distance (mm)		7.65 ± 1.9	10.84 ± 1.93	0.0001*

Values are given as the mean (standard deviation) and the p-value was calculated using the independent t test (*) and paired test (†).

## References

[1] Berninger MT, Hungerer S, Friederichs J, Stuby FM, Fulghum C (2019). Primary total hip arthroplasty in severe dysplastic hip osteoarthritis with a far proximal cup position. Journal of Arthroplasty.

[2] Nie Y, Pei F, Shen B, Kang P, Li Z (2015). Implication of acetabular width on the anteroposterior pelvic radiograph of patients with developmental dysplasia of the hip. Journal of Arthroplasty.

[3] Fujii M, Nakamura T, Hara T, Nakashima Y, Iwamoto Y (2015). Does radiographic coxa profunda indicate increased acetabular coverage or depth in hip dysplasia?. Clinical Orthopaedics and Related Research.

[4] Xu J, Qu X, Li H, Mao Y, Yu D (2017). Three-dimensional host bone coverage in total hip arthroplasty for Crowe types II and III developmental dysplasia of the hip. Journal of Arthroplasty.

[5] Ito H, Matsuno T, Minami A, Aoki Y (2003). Intermediate-term results after hybrid total hip arthroplasty for the treatment of dysplastic hips. The Journal of Bone & Joint Surgery.

[6] Zheng LL, Lin YY, Zhang XY, Ling QH, Liao WM (2019). Best bone of acetabulum for cup component placement in Crowe types I to III dysplastic hips: A computer simulation study. Chinese Medical Journal.

[7] Tözün IR, Beksaç B, Sener N (2007). Acta Orthopaedica et Traumatologica Turcica.

[8] Du Y, Fu J, Sun J, Zhang G, Chen J (2020). Acetabular bone defect in total hip arthroplasty for Crowe II or III developmental dysplasia of the hip: A finite element study. Biomed Research International.

[9] Wang C, Xiao H, Yang W, Wang L, Hu Y (2019). Accuracy and practicability of a patient-specific guide using acetabular superolateral rim during THA in Crowe II/III DDH patients: A retrospective study. Journal of Orthopaedic Surgery and Research.

[10] Pagnano W, Hanssen AD, Lewallen DG, Shaughnessy WJ (1996). The effect of superior placement of the acetabular component on the rate of loosening after total hip arthroplasty. The Journal of Bone & Joint Surgery.

[11] Johnston RC, Brand RA, Crowninshield RD (1979). Reconstruction of the hip. A mathematical approach to determine optimum geometric relationships. The Journal of Bone & Joint Surgery.

[12] Bicanic G, Delimar D, Delimar M, Pecina M (2009). Influence of the acetabular cup position on hip load during arthroplasty in hip dysplasia. International Orthopaedics.

[13] Shen J, Sun J, Ma H, Du Y, Li T (2020). High hip center technique in total hip arthroplasty for Crowe type II-III developmental dysplasia: Results of midterm follow-up. Orthopaedic Surgery.

[14] Kaneuji A, Sugimori T, Ichiseki T, Yamada K, Fukui K (2009). Minimum ten-year results of a porous acetabular component for Crowe I to III hip dysplasia using an elevated hip center. Journal of Arthroplasty.

[15] Watts CD, Martin JR, Fehring KA, Griffin WL (2018). Inferomedial hip center decreases failure rates in cementless total hip arthroplasty for Crowe II and III hip dysplasia. Journal of Arthroplasty.

[16] Nawabi DH, Meftah M, Nam D, Ranawat AS, Ranawat CS (2014). Durable fixation achieved with medialized, high hip center cementless THAs for Crowe II and III dysplasia. Clinical Orthopaedics and Related Research.

[17] Tetsunaga T, Fujiwara K, Endo H, Tetsunaga T, Miyake T (2019). Changes in acetabular component alignment due to screw fixation in patients with hip dysplasia. Hip International.

[18] Adler E, Stuchin SA, Kummer FJ (1992). Stability of press-fit acetabular cups. Journal of Arthroplasty.

[19] Kwong LM, O’Connor DO, Sedlacek RC, Krushell RJ, Maloney WJ (1994). A quantitative in vitro assessment of fit and screw fixation on the stability of a cementless hemispherical acetabular component. Journal of Arthroplasty.

[20] Spangehl MJ, Berry DJ, Trousdale RT, Cabanela ME (2001). Uncemented acetabular components with bulk femoral head autograft for acetabular reconstruction in developmental dysplasia of the hip: Results at five to twelve years. The Journal of Bone & Joint Surgery.

[21] Kim M, Kadowaki T (2010). High long-term survival of bulk femoral head autograft for acetabular reconstruction in cementless THA for developmental hip dysplasia. Clinical Orthopaedics and Related Research.

[22] Heller MO, Schröder JH, Matziolis G, Sharenkov A, Taylor WR (2007). Musculoskeletal load analysis. A biomechanical explanation for clinical results and more?. Orthopade.

[23] Dorr LD, Tawakkol S, Moorthy M, Long W, Wan Z (1999). Medial protrusio technique for placement of a porous-coated, hemispherical acetabular component without cement in a total hip arthroplasty in patients who have acetabular dysplasia. The Journal of Bone & Joint Surgery.

[24] Udomkiat P, Dorr LD, Wan Z (2002). Cementless hemispheric porous-coated sockets implanted with press-fit technique without screws: Average ten-year follow-up. The Journal of Bone & Joint Surgery.

[25] Schmalzried TP, Hill GC, Wessinger SJ, Harris WH (1994). The Harris_galante porous acetabular component press-fit without screw fixation: Five-year radiographic analysis of primary cases. Journal of Arthroplasty.

[26] Takao M, Nakamura N, Ohzono K, Sakai T, Nishii T (2011). The results of a press-fit-only technique for acetabular fixation in hip dysplasia. Journal of Arthroplasty.

[27] Ranawat CS, Dorr LD, Inglis AE (1980). Total hip arthroplasty in protrusio acetabuli of rheumatoid arthritis. The Journal of Bone & Joint Surgery.

[28] Crowe JF, Mani VJ, Ranawat CS (1979). Total hip replacement in congenital dislocation and dysplasia of the hip. The Journal of Bone & Joint Surgery.

[29] Charnley J, Feagin JA (1973). Low-friction arthroplasty in congenital subluxation of the hip. Clinical Orthopaedics and Related Research.

[30] Calaghan JJ, Dysart SH, Savory CG (1988). The uncemented porous-coated anatomic total hip prothesis. Two-year results of a prospective consecutive series. The Journal of Bone & Joint Surgery.

[31] Anderson MJ, Harris WH (1999). Total hip arthroplasty with insertion of the acetabular component without cement in hips with total congenital dislocation or marked congenital dysplasia. The Journal of Bone & Joint Surgery.

[32] Hartofilakidis G, Stamos K, Karachalios T (1998). Treatment of high dislocation of the hip in adults with total hip arthroplasty. Operative technique and long-term clinical results. The Journal of Bone & Joint Surgery.

[33] Sakemi Y, Komiyama K, Yoshimoto K, Shiomoto K, Iwamoto M (2019). How does anteroposterior cup placement affect bone coverage and range of motion in primary total hip arthroplasty for hip dysplasia?. Journal of Orthopaedic Science.

[34] Greber EM, Pelt CE, Gililland JM, Anderson MB, Erickson JA (2017). Challenges in total hip arthroplasty in the setting of developmental dysplasia of the hip. Journal of Arthroplasty.

[35] Holzapfel BM, Greimel F, Prodinger PM, Pilge H, Nöth U (2012). Total hip replacement in developmental dysplasia using an oval-shaped cementless press-fit cup. International Orthopaedics.

[36] Mulroy RD, Harris WH (1990). Failure of acetabular autogenous grafts in total hip arthroplasty. Increasing incidence: a follow-up note. The Journal of Bone & Joint Surgery.

[37] Nie Y, Wang H, Huang Z, Shen B, Kraus VB (2018). Radiographic underestimation of in vivo cup coverage provided by total hip arthroplasty for dysplasia. Orthopedics.

[38] Hirakawa K, Mitsugi N, Koshino T, Saito T, Hirasawa Y (2001). Effect of acetabular cup position and orientation in cemented total hip arthroplasty. Clinical Orthopaedics and Related Research.

[39] Tikhilov R, Shubnyakov I, Burns S, Shabrov N, Kuzin A (2016). Experimental study of the installation acetabular component with uncoverage in arthroplasty patients with severe developmental hip dysplasia. International Orthopaedics.

